# Anodal Transcranial Direct Current Stimulation Can Improve Spatial Learning and Memory and Attenuate Aβ_42_ Burden at the Early Stage of Alzheimer’s Disease in APP/PS1 Transgenic Mice

**DOI:** 10.3389/fnagi.2020.00134

**Published:** 2020-05-13

**Authors:** Yinpei Luo, Wenjuan Yang, Nian Li, Xiufang Yang, Binglian Zhu, Cong Wang, Wensheng Hou, Xing Wang, Huizhong Wen, Xuelong Tian

**Affiliations:** ^1^Chongqing Engineering Research Center for Medical Electronics Technology, Bioengineering College, Chongqing University, Chongqing, China; ^2^Department of Neurobiology, College of Basic Medical Science, Chongqing Key Laboratory of Neurobiology, Army Medical University, Chongqing, China; ^3^College of Microelectronics and Communication Engineering, Chongqing University, Chongqing, China

**Keywords:** transcranial direct current stimulation, Alzheimer’s disease, early intervention, spatial learning and memory, β-amyloid

## Abstract

Alzheimer’s disease (AD) is an irreversible progressive neurodegenerative disease. Intervention in the early stage of AD is a new path for AD treatment that is being explored. The behavioral and pathological effects of anodal transcranial direct current stimulation (AtDCS) at the early stage of AD in the mouse model, amyloid precursor protein (APP)/presenilin-1 (PS1) transgenic mice, were investigated based on our previous studies. Thirty-three 6-month-old male APP/PS1 mice were randomly divided into the model group (AD group), model + sham stimulation group (ADST group) and stimulation group (ADT group). Eleven 6-month-old male C57 wild-type mice were randomly selected as a control group (CTL group). The ADT group received 10 AtDCS sessions. The Morris water maze (MWM) task and novel object recognition (NOR) task were used to test mouse memory. Nissl staining, Western blot (WB), immunohistochemistry and immunofluorescence staining of β-amyloid (Aβ_42_), glial fibrillary acidic protein (GFAP) and NF200 were conducted for pathological analysis. The ADT group and the CTL group had a shorter escape latency and more platform-region crossings than the AD group and ADST group in the MWM. There was no significant difference in the discrimination index among the groups in the NOR task. Pathological analysis showed visible differences between the AD group and ADT group. This study revealed that early-stage APP/PS1 transgenic mice did not show recognition memory impairment. AtDCS effectively improved spatial learning and memory in the early-stage APP/PS1 transgenic mouse model of AD, alleviating Aβ burden and having a protective effect on neurons. AtDCS could improve AD-related symptoms by activating many glial cells to promote the degradation and clearance of Aβ or directly affecting production and degradation of Aβ to reduce glial activation. AtDCS is an effective means of early intervention in the early stage of AD.

## Introduction

Alzheimer’s disease (AD) is a serious neurodegenerative disease characterized by cognitive and memory dysfunction (Cummings et al., 2014; Alzheimer’s Association, 2018). It has become the seventh leading cause of death in the world (Patterson, 2018). AD begins decades before clinical symptoms appear, and it is a slowly changing, irreversible process that occurs over time (Sperling et al., 2011; Bateman et al., 2012; Dubois et al., 2016). Based on the long preclinical stage and the irreversible characteristics of AD, the clinical stage of AD may not be the optimal period of treatment (Selkoe and Hardy, 2016). The National Institute on Aging and the Alzheimer’s Association divide preclinical AD into three phases (Sperling et al., 2011): Stage 1, asymptomatic cerebral amyloidosis; Stage 2, neuron degeneration; Stage 3, subtle cognitive/behavioral decline, which eventually evolves into AD with time. Some researchers have gradually focused on the treatment of AD in the preclinical AD period. Early intervention before AD diagnosis may delay or even prevent brain lesions, thereby significantly reducing the symptoms of patients with AD and the cost of aged care (McManus and Heneka, 2017; Alzheimer’s Association, 2018).

Transcranial direct current stimulation (tDCS) is a noninvasive neuromodulation technique that delivers a constant low-intensity subthreshold direct current to specific regions of the brain through electrodes placed on the scalp, thereby regulating cell transmembrane potential depolarization and hyperpolarization (Bindman et al., 1962; Nitsche and Paulus, 2000) and altering neuronal activity and excitability of the cerebral cortex (Nitsche et al., 2008; Stagg et al., 2018). A number of clinical and basic studies have found that tDCS treatment can improve memory and cognitive dysfunction in patients and animal with AD (Yu et al., 2014; Hsu et al., 2015; Liu et al., 2017; Cruz Gonzalez et al., 2018). However, few studies have validated tDCS in the preclinical AD phase, and little is known about the mechanism of action.

The understanding of the mechanism of tDCS for AD is still in its infancy. There is currently no direct mechanism study on the application of tDCS to AD. In our previous studies, anodal tDCS (AtDCS) improved spatial learning and memory in an AD rat model, and the activity of astrocytes was significantly reduced (Yu et al., 2015), an effect that lasted for 2 months (Yang et al., 2019). AtDCS reduces the number of dysfunctional astrocytes and, in turn, reduces neurotoxicity, which may be the reason why AtDCS improves spatial learning and memory in AD rats. Herein, we performed AtDCS on early-stage amyloid precursor protein (APP)/presenilin-1 (PS1) transgenic AD mice, observed the resulting behavioral and pathological changes, and explored the mechanism of AtDCS in early-stage AD.

## Materials and Methods

### Study Design

**Figure 1:**
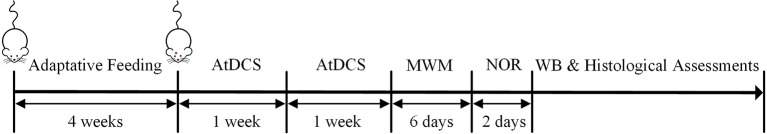
Timeline of the experiment. After 4 weeks of adaptation of the four groups to the feeding conditions, 10 AtDCS sessions were performed on the ADT group. Then, all four groups of mice were subjected to the morris water maze (MWM) and novel object recognition (NOR) tasks in sequence. Finally, Western blot (WB) and histological assessments were performed.

This randomized and double-blind animal study was approved by the Laboratory Animal Welfare and Ethics Committee of the Army Medical University. The 5-month-old APPswe/PSEN1dE9 (APP/PS1) transgenic mice (male, weight: 18–28 g, Institute of Animal Models, Nanjing) and C57 wild-type mice (male, weight: 18–28 g) were adapted to feeding conditions for 4 weeks. Water and food were freely available, and mice were housed on a 12-h light/dark cycle at room temperature (24 ± 1°C). The APP/PS1 transgenic mice were randomly divided into three groups: AD group, ADST group, and ADT group, and C57 wild-type mice were used as the control (CTL) group, with 11 mice in each group. One day before the AtDCS, electrodes were placed in the mice in each group. After 10 AtDCS sessions in the ADT group, each group of mice was subjected to the Morris water maze (MWM) and novel object recognition (NOR) tasks in sequence, followed by Western blot (WB) and histological assessments. The timeline of the experiment is shown in Figure 1. All animal experiments described in this study were implemented according to the Guide for the Care and Use of Laboratory Animals of National Institutes of Health in the USA.

### Anodal Transcranial Direct Current Stimulation

AtDCS was initiated after four groups of mice underwent 4 weeks of adaptive feeding. At this time, all four groups of mice were 6 months of age. Based on our previous protocol (Yu et al., 2015; Yang et al., 2019), electrodes were installed in APP/PS1 mice 1 day prior to stimulation. The anode electrode was a cylindrical plastic tube made of polyvinyl chloride, and was filled with a sponge and an outer guiding copper wire (diameter: 2 mm), the effective contact area of which was 3.14 mm^2^. The cathode electrode was a circular silver chloride ECG electrode (diameter: 2 cm) with an effective contact area of 3.14 cm^2^. The anode electrode was fixed on the skull over the frontal cortex of the mice by glass ionomer cement (Medical Devices Co., Ltd. Shanghai), and the cathode electrode was placed in the chest and abdomen. The anode electrode and cathode electrode were wetted with physiological saline to reduce the contact resistance and ensure good electrical conductivity of the circuit before stimulation. Twenty-four hours after electrode installation, the ADT group was subjected to AtDCS. The stimulation intensity of a single AtDCS session was 150 μA, and the stimulation duration was 30 min. An AtDCS session was performed on the ADT group every day for 5 days, followed by a 2-day rest period. The ADT group was subjected to two courses of treatment. During the stimulation process, the current intensity was monitored by a multimeter at all times. Sham stimulation (AtDCS, 150 μA, 10 s) was performed in the ADST group. Notably, mice were not anesthetized during AtDCS or sham AtDCS.

### Morris Water Maze

After AtDCS, the spatial learning and memory ability of the mice were assessed by MWM. The MWM test was performed in a white circular pool (diameter: 120 cm) filled with water equilibrated to room temperature (22°C). The pool was divided into four quadrants according to the Cartesian coordinate system. Each quadrant of the pool wall was labeled with different colors and shapes, and these labels remained unchanged during the experiment. A circular platform (diameter: 12 cm) was placed in the middle of the third quadrant (Bromley-Brits et al., 2011).

The MWM task contained three tests: the visible platform test, hidden platform test and probe test. The visible platform test was performed on the first day of the MWM task. The circular platform extended 1 cm above the water surface. The mice were placed into the pool at different quadrant walls. The time that the mouse took to find and board the platform within 60 s was recorded as the escape latency. If the mouse did not find the platform within 60 s, the mice were guided to the platform and left on the platform for 5 s, and the escape latency was recorded as 60 s. At the same time, the path length of the mouse before escaping to the visible platform was recorded. If the escape latency and path length were not significantly different among groups, the mice in each group were considered to have similar motor and visual abilities. The second to fifth day of the MWM task was the hidden platform test. The circular platform extended 1 cm below the water surface, and the different quadrants were used as entry points. The mice were placed gently into the water, facing the edge of the pool, and the escape latency and the path length before the mice escaped to the hidden platform within 60 s were recorded. The mice underwent four sessions a day for 4 days with a 20-min interval between each training session. The sixth day of the MWM task was the probe test. The platform was removed, and the mice were placed in the water in the first quadrant. The number of platform-region crossings within 30 s and the time spent in each quadrant were recorded.

### Novel Object Recognition

After the end of the MWM, all mice were subjected to the NOR task to assess the recognition memory of the mice. The NOR task utilized a white test box (25 cm * 25 cm * 32 cm), two yellow cuboids (2 cm * 2 cm * 4 cm), and a red cone (2 cm * 2 cm * 4 cm; Bevins and Besheer, 2006).

**Figure 2:**
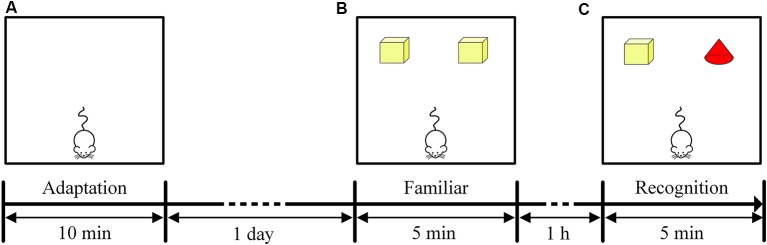
Timeline of the NOR task. **(A)** Experimental design of the adaptation period in the NOR task. **(B)** Experimental design of the familiar period in the NOR task. **(C)** Experimental design of the recognition period in the NOR task.

The NOR task included three stages: an adaptation period, familiar period and recognition period. The adaptation period occurred on the first day of the NOR task. The mice were placed in the test box, and each mouse was allowed to move freely for 10 min to adapt to the environment (Figure 2A). The familiarization period and recognition period occurred on the second day of the NOR task. During the familiarization period, two yellow cuboids were placed in the test box, and each mouse was allowed to freely explore the object for 5 min (Figure 2B). The recognition period then began 1 h after the familiarization period. One of the yellow cuboids was replaced with a red cone, and each mouse was allowed to freely explore the object for 5 min (Figure 2C). The time that the mice spent exploring each of the two objects was recorded separately (recorded as the time that the mice approached within 2 cm of an object). The recognition memory of the mice was assessed by the discrimination ratio [during the recognition period, the time the mouse explored the novel object/(the sum of the time spent exploring the familiar and novel objects) × 100%]. A discrimination ratio above 50% indicated good recognition performance. At the end of each test, the test box was wiped with alcohol to remove the odor left by the mice. The test environment was soundproof and protected from light, and there was no shadow in the test box.

### Western Blot

WB was carried out as described by Zhao et al. (2018) and Sun et al. (2019a). On the second day after the NOR task, the hippocampus was quickly removed from four groups of mice (*n* = 3). Proteins from the mouse hippocampus were extracted using a protein extraction kit (Beyotime Biotech), and the protein concentration was determined using a BCA analysis kit (Beyotime Biotech). The protein sample was diluted in loading buffer, and sodium dodecyl sulfate polyacrylamide gel electrophoresis (SDS-PAGE) was used to separate the same amount of protein, which was then transferred to a PVDF membrane. After the transfer was completed, the membrane was blocked for 2 h at room temperature in a 5% Protein Blocking (Boster) Solution configured with Tris-Buffer Saline Tween 20 (TBST). PVDF membranes were trimmed according to the molecular weight of the prestained marker and the protein of interest and incubated with the corresponding primary antibodies [Anti-GAPDH, anti-Aβ_42_, anti-glial fibrillary acidic protein (GFAP), anti-NF200; 1:1,000] at 4°C overnight. The next day, the PVDF membrane was rinsed five times in TBST buffer for 8 min each. The membrane was placed in a solution containing secondary antibodies (Goat anti-mouse, goat anti-rabbit; 1:2,000) at 37°C for 1.5 h and then rinsed five times in TBST buffer for 8 min each rinse. The blots were finally visualized with chemiluminescent HRP substrate (Millipore) for 1 min by Western Lightning-ECL. The experiments were repeated at least three times. The blots were placed in a gel imager (Bio-Rad), and the optical density of each band was measured using Quantity One software (Bio-Rad) and normalized to that of GAPDH. Information on the antibodies is displayed in Table 1.

**Table 1:** Information on the antibodies used in this study.

	Antibodies	Host species	Company	Cat No.
Primary antibodies	Anti-GAPDH	Rabbit	Beyotime, China	AF1186
	Anti-Aβ_42_	Rabbit	Abcam, United Kingdom	ab201060
	Anti-GFAP	Mouse	Cell Signaling Technology, United States	3670S
	Anti-NF200	Mouse	Abcam, United Kingdom	ab82259
Secondary antibodies	Goat anti-mouse (H+L)	-	Zhongshan Goldenbridge Biotechnology, China	ZB-2305
	Goat anti-rabbit (H+L)	-	Zhongshan Goldenbridge Biotechnology, China	ZB-2301
	Anti-mouse IgG (H+L), Alexa Fluor 594	Goat	Cell Signaling Technology, United States	8890S
	Anti-rabbit IgG (H+L), Alexa Fluor 488	Goat	Cell Signaling Technology, United States	4412S

### Histological Assessments

On the second day after the behavioral tasks, mice (*n* = 8) from three groups (CTL group, AD group, and ADT group) were deeply anesthetized and perfused with 0.9% physiological saline solution. Then, the mice were fixed in 4% paraformaldehyde for 1 h, and the brains of the mice were quickly removed. The brain was soaked in 4% paraformaldehyde overnight at 4°C and then transferred to a 30% sucrose solution at 4°C until sank to the bottom. Frozen mouse brain coronal sections with a thickness of 30 μm were obtained with a freezing microtome.

#### Nissl Staining

Brain sections were washed three times with 1% PBS for 5 min each time. Then, brain sections were stained in 1% toluidine blue solution for 2 min and washed twice with 1% PBS for 5 min each time. All brain sections were mounted on glass slides, dried in a 37°C incubator, dehydrated in an alcohol gradient and clarified in xylene. The sections were covered with a neutral gum, and the slides were stored in a cool, well-ventilated place.

#### Immunohistochemistry

First, brain sections were washed three times with 1% PBS for 5 min each time. The washed brain sections were incubated with 10% goat serum at 37°C for 30 min in an incubator. Then, the brain sections were incubated separately in a solution containing primary antibodies (Anti-Aβ_42_, anti-GFAP, anti-NF200; 1:200) at 37°C for 1 h and overnight at 4°C. After the overnight inclubation, brain sections were washed three times in 1% PBS for 5 min each time and then placed separately in a solution containing secondary antibodies (Goat anti-mouse, goat anti-rabbit; 1:500) at 37°C for 1 h. The brain sections were then washed three times in 1% PBS for 5 min each time and were visualized with DAB-enhanced color development solution for 5 min. All brain sections were mounted on glass slides, dried in a 37°C incubator, dehydrated in an alcohol gradient and clarified in xylene. The sections were covered with a neutral gum, and the slides were stored in a cool, well-ventilated place. Information on the antibodies is displayed in Table 1.

#### Immunofluorescence

The immunofluorescence procedure was the same as the immunohistochemical procedure before incubating the primary antibody. Then, the brain sections were incubated in a solution containing primary antibodies (Anti-Aβ_42_, anti-GFAP; 1:1,000) at 37°C for 1 h and overnight at 4°C. After the overnight incubation, the brain sections were washed three times in 1% PBS for 5 min each time and then placed in a solution containing fluorescent secondary antibodies (Anti-mouse IgG, anti-rabbit IgG; 1:1,000) at 37°C for 1 h. Brain sections were placed in 4′,6-diamidino-2-phenylindole (DAPI, Sigma) for 10 min and then washed three times in 1% PBS for 5 min each time. All brain sections were mounted on glass slides with a Fluoromount-G fluorescent seal (Southern Biotech) and saved in a cassette. Information on the antibodies is displayed in Table 1.

### Images

The images were acquired with a DP70 digital camera equipped with an Olympus microscope (image resolution: 1,360 × 1,024, Olympus Co. Ltd., Japan). The 3D immunofluorescence images were acquired by Axio Observer.Z1 Inverted Microscope with LD Plan-Neofluar objective (image resolution: 1,025 × 1,025, ZEISS Co. Ltd., Germany). Images of histological staining of mice in the three groups were obtained using the same Olympus microscope. Microscope adjustments were made for rotation, brightness and contrast, and choices on the size and tailoring of the image were made. The number and area fraction of Aβ_42_, the integrated optical density (IOD) of GFAP- and NF200- positive immunoresponsive cells were quantitatively measured by Image Pro Plus 6.0 software. The area fraction was reported as the percentage of total hippocampal or frontal cortex area containing Aβ_42_.

### Statistical Analysis

The statistical results of the data were expressed as the mean ± standard error of the mean (SEM). Data processing was performed using IBM SPSS Statistics 25.0, and Tukey’s test was used as a *post hoc* test. *P* < 0.05 was considered statistically significant. The statistical power (power) of the behavioral experiments was calculated by G*Power 3.1.9.2 software with an α-error probability value of 0.05. In the MWM, repeated measures analysis of variance (ANOVA) was used to analyze the escape latency of the four groups of mice in the hidden platform experiment, and one-way ANOVA was used to analyze the escape latency in the visible platform experiment and the number of platform-region crossings and the time spent in each quadrant in the probe test. In the NOR, one-way ANOVA was used to analyze the discrimination ratio of the four groups. For the WB and histological assessments, the levels of Aβ_42_, GFAP and NF200, the number and area fraction of Aβ_42_ deposits and the IOD of GFAP- and NF200-positive immunoresponsive cells were analyzed using one-way ANOVA.

## Results

All 11 APP/PS1 transgenic mice in the ADT group were well treated with AtDCS and performed behavioral tasks with 33 other mice in the CTL, ADST, and AD groups.

### AtDCS Improves Spatial Learning and Memory in Early-Stage APP/PS1 Transgenic Mice

**Figure 3:**
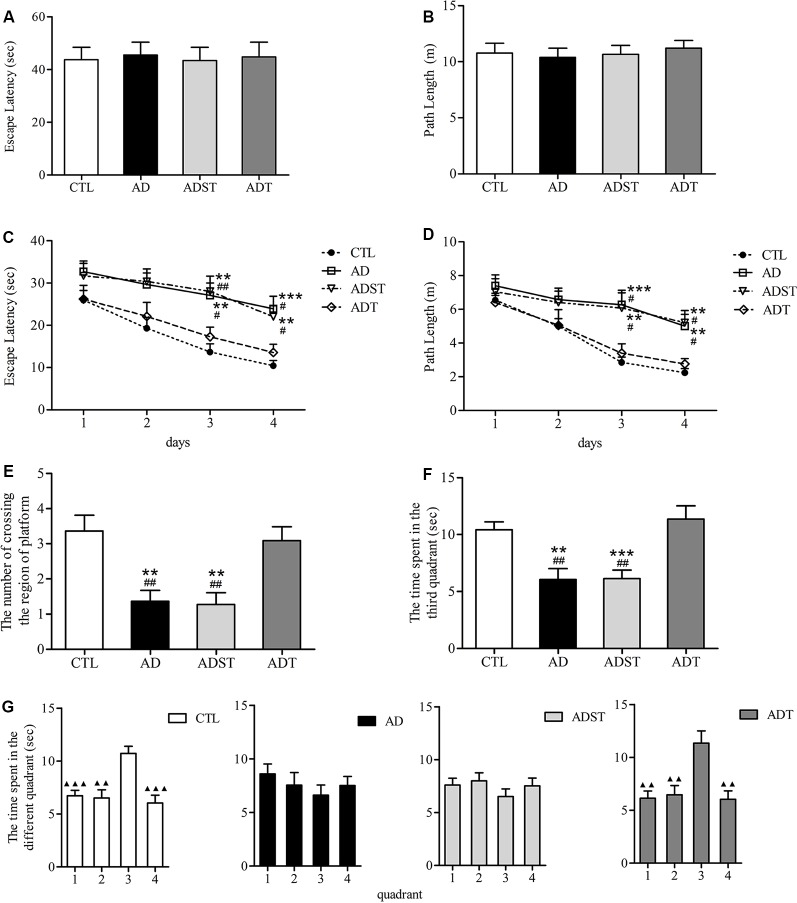
Effects of AtDCS on spatial learning and memory of 6-month-old APP/PS1 transgenic mice. **(A,B)** Latency and path length of the mice in the four groups escaping to the visible platform in the visible platform test. **(C,D)** The latency and path length of the mice in the four groups escaping to the hidden platform in the hidden platform test. **(E,F)** In the probe test, the number of platform-region crossings and the time spent in the third quadrant of the mice in the four groups. **(G)** In the probe test, the time spent in each quadrant in the four groups of mice. 1, the first quadrant; 2, the second quadrant; 3, the third quadrant; 4, the fourth quadrant. All data are presented as the means ± SEM. *n* = 11 for each group. Alzheimer’s disease (AD), ADST, and ADT vs. CTL: **P < 0.005, ***P < 0.001. AD, ADST vs. ADT: ^#^*P* < 0.05, ^##^*P* < 0.005. The first, second, and fourth quadrant vs. the third quadrant, ^▴^*P* < 0.005, ^▴▴^*P* < 0.001.

In the visible platform test, there were no differences among the mice of the four groups in the latency (Figure 3A, *F*_(3,172)_ = 0.021, *P* = 0.996, power = 0.053) or the path length (Figure 3B, *F*_(3,172)_ = 0.181, *P* = 0.910, Power = 0.168) to escape to the visible platform. All mice had similar motor and visual abilities, and there was no case where mice were unable to find the platform. Therefore, all mice participated in the follow-up procedure of the MWM. In the hidden platform test, repeated measures ANOVA showed a significant main effect of group (escape latency, *F*_(3,172)_ = 14.641, *P* < 0.001, power = 0.892; path length, *F*_(3,172)_ = 11.133, *P* < 0.001, power = 0.851) and day (escape latency, *F*_(3,172)_ = 13.774, *P* < 0.001, power = 0.832; path length, *F*_(3,172)_ = 14.194, *P* < 0.001, power = 0.866) on the escape latency and the path length. However, there was no significant difference on the day × group interaction (escape latency, *F*_(9,172)_ = 0.407, *P* = 0.930, power = 0.082; path length, *F*_(9,172)_ = 1.496, *P* = 0.153, power = 0.217). the escape latency (Figure 3C, AD, day 3, *P* = 0.001, day 4, *P* < 0.001; ADST, day 3, *P* = 0.003, day 4, *P* = 0.004) and the path length (Figure 3D, day 3, *P* < 0.001, day 4, *P* = 0.004; ADST, day 3, *P* = 0.002, day 4, *P* = 0.001) of the AD group and the ADST group were significantly different from those of the CTL group. The escape latency (Figure 3C, AD, day 3, *P* = 0.005, day 4, *P* = 0.005; ADST, day 3, *P* = 0.003, day 4, *P* = 0.006) and path length (Figure 3D, AD, day 3, *P* = 0.007, day 4, *P* = 0.009; ADST, day 3, *P* = 0.008, day 4, *P* = 0.019) of the ADT group were significantly different from those of the AD group and ADST group. In the probe test, the number of platform-region crossings (Figure 3E, AD, *F*_(1,20)_ = 13.297, *P* = 0.002, power = 0.844; ADST, *F*_(1,20)_ = 13.848, *P* = 0.001, power = 0.874) and time spent in the third quadrant (Figure 3F, AD, *F*_(1,20)_ = 13.688, *P* = 0.001, power = 0.988; ADST, *F*_(1,20)_ = 17.719, *P* < 0.001, power = 0.986) of the AD group and the ADST group were significantly different from those of the CTL group. Compared with the AD group and the ADST group, the ADT group traversed the platform region significantly more times (Figure 3E, AD, *F*_(1,20)_ = 11.954, *P* = 0.002, power = 0.963; ADST, *F*_(1,20)_ = 12.500, *P* = 0.002, power = 0.956) and spent significantly more time in the third quadrant (Figure 3F, AD, *F*_(1,20)_ = 12.261, *P* = 0.002, power = 0.961; ADST, *F*_(1,20)_ = 14.111, *P* = 0.001, power = 0.997). Among the four groups of mice, only the CTL and ADT groups exhibited significant differences between the time spent in the third quadrant and the time spent in the other quadrants (Figure 3G, the first, second, and fourth quadrant vs. the third quadrant: CTL, *F*_(1,20)_ = 22.029, 16.904, 22.056, *P* < 0.001, = 0.001, <0.001, power = 0.999, 0.957, 0.997; ADT, *F*_(1,20)_ = 15.119, 11.338, 14.339, *P* = 0.001, 0.003, 0.001, power = 0.999, 0.962, 0.871). These results indicated that the CTL and ADT groups effectively distinguished the quadrant in which the platform was located from the other quadrants. In the MWM, there were no statistically significant differences between the ADT group and the CTL group or between the AD group and the ADST group (Figure 3). These data indicated that AtDCS treatment improved spatial learning and memory performance in early-stage APP/PS1 transgenic mice.

### Six-Month-Old APP/PS1 Transgenic Mice Do Not Show Recognition Memory Impairment

**Figure 4:**
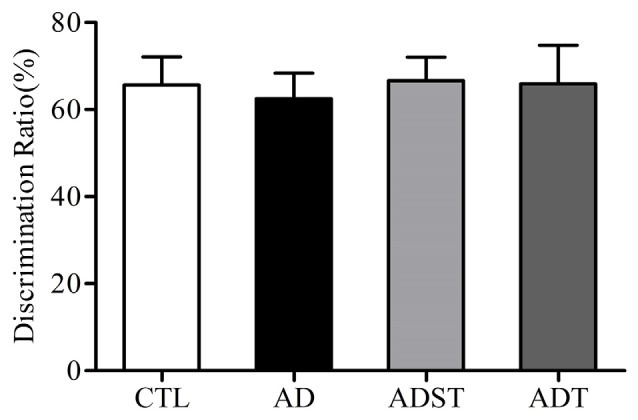
Effects of AtDCS on recognition memory of 6-month-old APP/PS1 transgenic mice. At 6 months of age, APP/PS1 transgenic mice did not exhibit recognition memory impairment (*P* > 0.05). All data are presented as the means ± SEM. *n* = 11 for each group.

The discrimination ratio of all the mice was higher than 50%. Compared to the discrimination ratios of the AD group (62.50 ± 5.84%) and the ADST group (66.66 ± 5.40%), the discrimination ratios of the ADT group (65.91 ± 8.83%) and the CTL group (65.65 ± 6.48%) were not significantly different (*F*_(3,40)_ = 0.074, *P* = 0.974, power = 0.062). There was also no significant difference in the discrimination ratio between the ADT group and the CTL group (Figure 4, *F*_(1,20)_ = 0.001, *P* = 0.981, power = 0.050). These results showed that APP/PS1 transgenic mice did not show recognition memory impairment at 6 months of age.

### AtDCS Altered the Protein Content of Related Molecules in Early-Stage APP/PS1 Transgenic Mice

**Figure 5:**
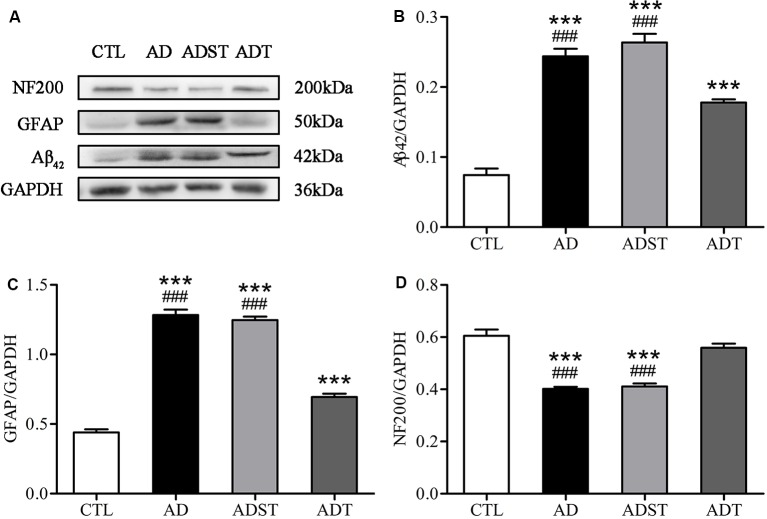
Effects of AtDCS on the levels of Aβ_42_, glial fibrillary acidic protein (GFAP), and NF200 in 6-month-old APP/PS1 transgenic mice. **(A)** Western blot results of Aβ_42_, GFAP, and NF200 expression in hippocampal tissue from each group. **(B)** Quantitative analyses of Aβ_42_ expression in hippocampal tissue from each group. **(C)** Quantitative analyses of GFAP expression in hippocampal tissue from each group. **(D)** Quantitative analyses of NF200 expression in hippocampal tissue from each group. All data are presented as the means ± SEM. *n* = 3 for each group. AD, ADST, and ADT vs. CTL: ***P < 0.001. AD, ADST vs. ADT: ^###^*P* < 0.001.

Aβ oligomers are one of the major neuropathological markers of AD. GFAP is a skeletal protein of astrocytes and is recognized as a characteristic astrocytic marker. NF200 is an intermediate filament found in the cytoplasm of neurons that is widely used to label neurons. WB results showed that compared with the AD and ADST group, the CTL group (vs. AD, Aβ_42_, *F*_(1,16)_ = 141.876, GFAP, *F*_(1,16)_ = 361.642, NF200, *F*_(1,16)_ = 66.025; vs. ADST, Aβ_42_, *F*_(1,16)_ = 153.697, GFAP, *F*_(1,16)_ = 548.100, NF200, *F*_(1,16)_ = 54.372; all *P* < 0.001) and ADT group (vs. AD, Aβ_42_, *F*_(1,16)_ = 609.422, GFAP, *F*_(1,16)_ = 174.968, NF200, *F*_(1,16)_ = 78.230; vs. ADST, Aβ_42_, *F*_(1,16)_ = 559.801, GFAP, *F*_(1,16)_ = 253.792, NF200, *F*_(1,16)_ = 57.307; all *P* < 0.001) exhibited significant differences in Aβ_42_, GFAP, and NF200 levels in the hippocampus. Compared with the CTL group, the ADT group had significant differences in Aβ_42_ and GFAP levels in the hippocampus (Aβ_42_, *F*_(1,16)_ = 941.085, GFAP, *F*_(1,16)_ = 59.810; all *P* < 0.001), but no significant difference in NF200 levels (NF200, *F*_(1,16)_ = 2.582, *P* = 0.128; Figure 5). These results indicated that AtDCS reduced the levels of Aβ_42_ and GFAP and increased the levels of NF200 in early-stage APP/PS1 transgenic mice.

### Histological Assessments

#### AtDCS Reduces Aβ_42_ Levels in Early-Stage APP/PS1 Transgenic Mice

**Figure 6:**
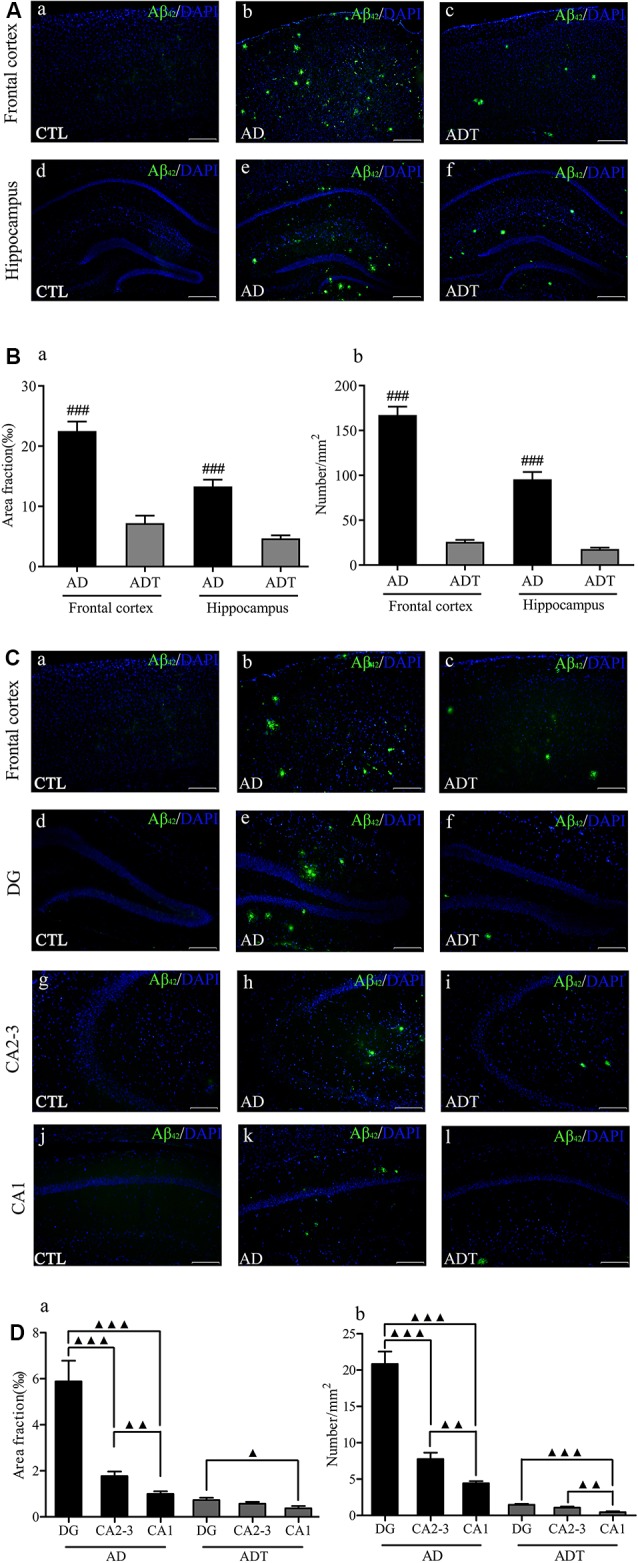
Effect of AtDCS on Aβ_42_ burden in 6-month-old APP/PS1 transgenic mice. **(A)** Aβ_42_ immunofluorescence staining results of each group in the frontal cortex and hippocampus. Scale bar, 500 μm. **(B)** Comparison of the area fraction and number of Aβ_42_ deposits of each group in the frontal cortex and hippocampus. **(C)** Aβ_42_ immunofluorescence staining results of each group in the frontal cortex and hippocampus. Scale bar, 200 μm. **(D)** Comparison of the area fraction and number of Aβ_42_ deposits in different regions of the hippocampus of the AD and ADT group. All data are presented as the means ± SEM. *n* = 8 for each group. AD vs. ADT: ^###^*P* < 0.001. Others, ^▴^*P* < 0.05, ^▴^*P* < 0.005, ^▴▴^*P* < 0.001.

No Aβ_42_ was found in the CTL group, and no fluorescence occurred. Aβ_42_ appeared in the AD group and the ADT group (Figure 6A). The area fraction (Figure 6Ba, frontal cortex, *F*_(1,62)_ = 57.394; hippocampus, *F*_(1,62)_ = 50.058; all *P* < 0.001) and number (Figure 6Bb, frontal cortex, *F*_(1,62)_ = 216.619; hippocampus, *F*_(1,62)_ = 87.674; all *P* < 0.001) of Aβ_42_ deposits in the frontal cortex and hippocampus were significantly lower in the ADT group than in the AD group. The results indicated that AtDCS significantly reduced the level of Aβ_42_. Furthermore, the AD group and ADT group had similar Aβ plaque distribution patterns in different regions of the hippocampus. In the hippocampus of the AD and ADT groups, the level of Aβ_42_ was highest in the dentate gyrus (DG) region, followed by the CA2-3 region and the CA1 region (Figure 6C). Compared with the area fraction and number of Aβ_42_ deposits in the DG region, those in the CA2-3 (Figure 6Da, area fraction, *F*_(1,62)_ = 19.959, *P* < 0.001; Figure 6Db, number, *F*_(1,62)_ = 46.001, *P* < 0.001) and CA1 (Figure 6Da, area fraction, *F*_(1,62)_ = 29.098, *P* < 0.001; Figure 6Db, number, *F*_(1,62)_ = 88.574, *P* < 0.001) regions were significantly lower in the AD group, and those in the CA1 region were significantly lower than those in the CA2-3 region (Figure 6Da, area fraction, *F*_(1,62)_ = 9.584, *P* = 0.003; Figure 6Db, number, *F*_(1,62)_ = 11.103, *P* = 0.001). In the ADT group, the area fraction (Figure 6Da, *F*_(1,62)_ = 6.392, *P* = 0.014) and number (Figure 6Db, *F*_(1,62)_ = 32.219, *P* < 0.001) of Aβ_42_ deposits in the DG region were significantly higher than those in the CA1 region, and a significant difference in the number of Aβ_42_ deposits was also apparent between the CA2-3 region and the CA1 region (Figure 6Db, *F*_(1,62)_ = 11.567, *P* = 0.001). This distribution pattern may indicate that Aβ_42_ levels gradually decrease according to the direction of neuronal information transmission in the hippocampal circuit.

#### AtDCS Reduces Astrocytes in Early-Stage APP/PS1 Transgenic Mice

**Figure 7:**
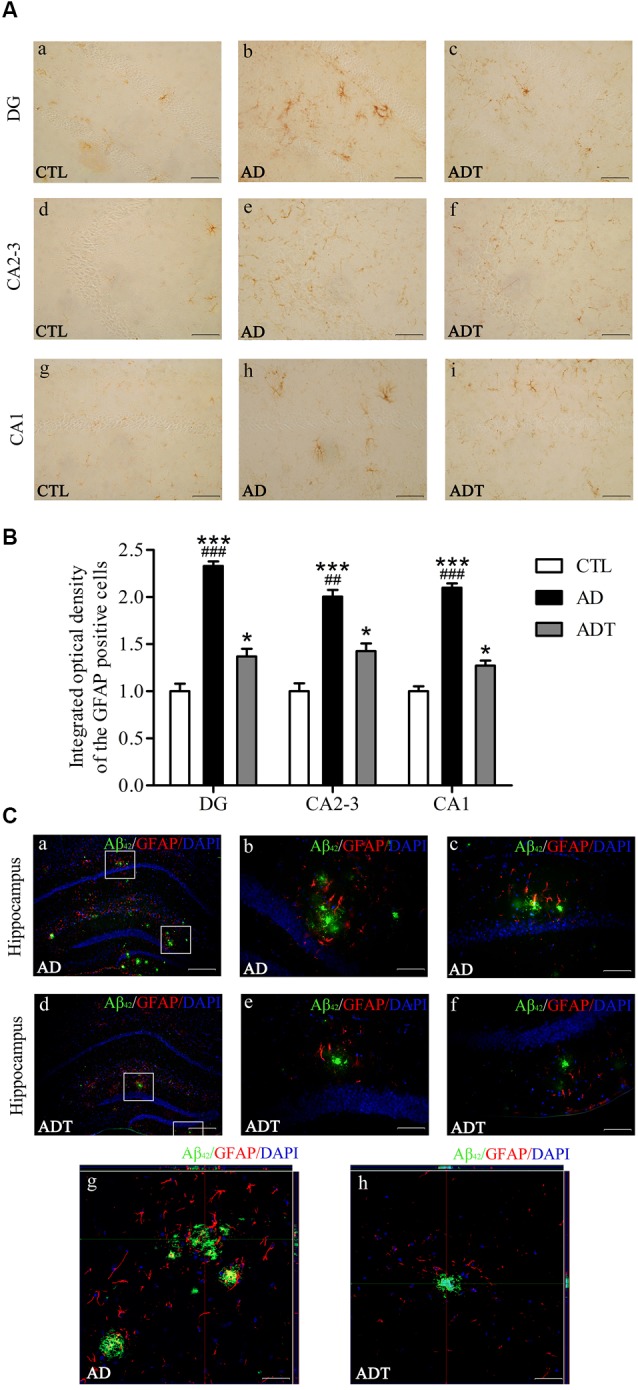
Effect of AtDCS on GFAP expression in 6-month-old APP/PS1 transgenic mice. **(A)** Results of GFAP immunohistochemical staining of the hippocampus in each group. Scale bar, 200 μm. **(B)** Comparison of integrated optical density (IOD) of hippocampal GFAP in each group. **(C)** Colabeling results of Aβ_42_ and GFAP immunofluorescence staining in the hippocampus of each group. **(a,d)** XY lateral view. Scale bar, 500 μm. **(b,c,e,f)** XY lateral view. Scale bar, 50 μm. **(g,h)** XY, XZ, and YZ lateral views. Scale bar, 50 μm. All data are presented as the means ± SEM. *n* = 8 for each group. AD, ADT vs. CTL: *P < 0.05, ***P < 0.001. AD vs. ADT: ^##^*P* < 0.005, ^###^*P* < 0.001.

Compared with the CTL group, the AD and ADT groups expressed significantly more GFAP (Figure 7B, AD: DG, *F*_(1,62)_ = 93.209, *P* < 0.001; CA2-3, *F*_(1,62)_ = 38.885, *P* < 0.001; CA1, *F*_(1,62)_ = 98.137, *P* < 0.001; ADT: DG, *F*_(1,62)_ = 4.284, *P* = 0.043; CA2-3, *F*_(1,62)_ = 4.591, *P* = 0.036; CA1, *F*_(1,62)_ = 4.216, *P* = 0.044), but the difference between the AD group and CTL group was more significant than that between the ADT group and CTL group. The expression level of GFAP in the ADT group (Figure 7B, DG: *F*_(1,62)_ = 36.518, *P* < 0.001; CA2-3: *F*_(1,62)_ = 9.187, *P* = 0.004; CA1: *F*_(1,62)_ = 47.650, *P* < 0.001) was significantly lower than that of the AD group. The results indicated that AtDCS significantly reduced the expression level of GFAP. Aβ_42_ and GFAP were colocalized in the AD group and the ADT group, with Aβ_42_ deposits surrounded by GFAP. GFAP was more highly expressed in areas with more plaques (Figure 7C).

#### AtDCS has Neuron-Protective Effects in Early-Stage APP/PS1 Transgenic Mice

**Figure 8:**
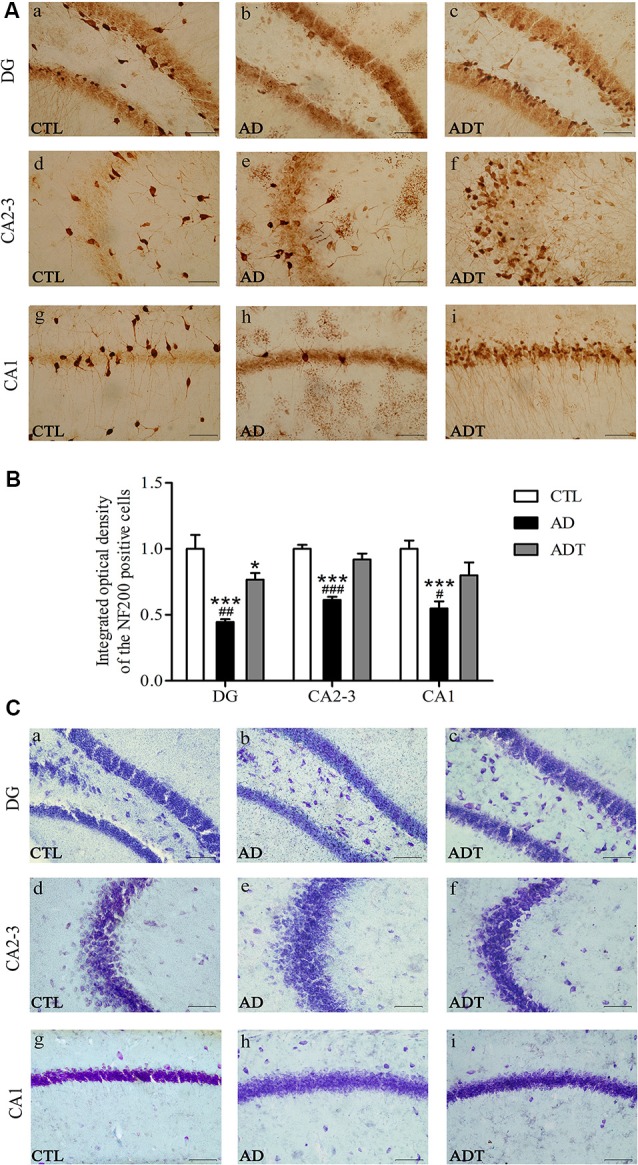
Effects of AtDCS on neurons in 6-month-old APP/PS1 transgenic mice. **(A)** NF200 immunohistochemical staining results of the hippocampus in each group. Scale bar, 200 μm. **(B)** Comparison of IOD of hippocampal NF200 in each group. **(C)** Nissl staining results of the hippocampus in each group. Scale bar, 200 μm. All data are presented as the means ± SEM. *n* = 8 for each group. AD, ADT vs. CTL: *P < 0.05, ***P < 0.001. AD vs. ADT: ^#^*P* < 0.05, ^##^*P* < 0.005, ^###^*P* < 0.001.

Neurons in the CTL and ADT groups were dark and intact, whereas neuron damage in the AD group was severe (Figure 8A). Compared with NF200 expression in the CTL group, NF200 expression was significantly lower in the AD group (Figure 8B, DG: *F*_(1,62)_ = 27.590; CA2-3: *F*_(1,62)_ = 133.4; CA1: *F*_(1,62)_ = 41.961, all *P* < 0.001) and significantly different in the DG region of the ADT group (*F*_(1,62)_ = 4.228, *P* = 0.044), but there was no significant difference between NF200 expression in the CTL and ADT groups in the CA2-3 or CA1 region (CA2-3: *F*_(1,62)_ = 1.953, *P* = 0.168; CA1: *F*_(1,62)_ = 3.524, *P* = 0.065). The expression level of NF200 was significantly higher in the ADT group than in the AD group (Figure 8B, DG: *F*_(1,62)_ = 9.121, *P* = 0.004; CA2-3, *F*_(1,62)_ = 49.457, *P* < 0.001; CA1, *F*_(1,62)_ = 10.795, *P* = 0.017). Nissl staining was used to detect detect the morphological integrity of nerve cells. Compared with the neurons of the CTL group, the neurons of the AD group in the hippocampus were sparsely arranged, with light Nissl body staining. Compared with the neurons of the AD group, the neurons of the ADT group in the hippocampus were arranged more neatly, with darker and more obvious Nissl bodies (Figure 8C). NF200 immunohistochemistry and Nissl staining showed that AtDCS had neuron-protective effects in early-stage APP/PS1 transgenic mice.

## Discussion

The study evaluated the effects of AtDCS on preclinical AD and explored the mechanism of action of AtDCS in the early stages of AD. In this study, we demonstrated that AtDCS improved spatial learning and memory in 6-month-old APP/PS1 transgenic mice and slowed the progression of the AD pathological marker, Aβ, reducing the expression level of GFAP and neuronal damage with neuroprotective effects. The results suggest that the AtDCS technique is worth investigating; this method should be a therapeutic option for the early-stage AD based on rodent studies. In addition, we found that 6-month-old APP/PS1 transgenic mice did not show recognition memory impairment. The colocalization of GFAP with Aβ suggested that the mechanism of action of AtDCS in this stage of AD may have two pathways: AtDCS could promote the degradation and clearance of Aβ by activating many glial cells at an early stage; AtDCS may directly affect production and degradation of Aβ, and reduce the number of Aβ deposits, thereby reduce the activation of glial cells.

APP-PS1 transgenic mice are currently used as AD transgenic animal models. APP-PS1 transgenic mice mimic the progression of AD patients, and steady amyloid deposition is observed at 6 months of age in these mice (Jankowsky et al., 2004). Therefore, 6-month-old APP/PS1 transgenic mice can be used as an AD mouse model for experimental studies in the preclinical AD stage (Xiong et al., 2011). For APP/PS1 transgenic mice, spatial memory defects occur earlier than defects in recognition memory (Webster et al., 2013). APP/PS1 transgenic mice have been shown to have significant spatial memory deficits at 6 months of age, whereas behavioral abnormalities in recognition begin at approximately 30 weeks of age (Woo et al., 2010). In our behavioral studies, AtDCS treatment significantly improved the spatial learning and memory abilities of 6-month-old APP/PS1 transgenic mice, as assessed by the MWM, which is consistent with previous studies (Woo et al., 2010; Webster et al., 2013). At the same time, 6-month-old APP/PS1 transgenic mice did not show recognition memory impairment evaluated by the NOR task.

Amyloid positivity goes through three stages of preclinical AD (Sperling et al., 2011). The amyloid cascade hypothesis is one of the hypotheses of AD pathology and assumes that neurodegeneration in AD pathology is caused by abnormal aggregation of Aβ to form Aβ oligomers (Hardy and Higgins, 1992; Barage and Sonawane, 2015). Of the Aβ oligomer isoforms formed, Aβ_40_ is the most common, followed by Aβ_42_. However, Aβ_42_ is hydrophobic and aggregates at a faster rate than Aβ_40_ (Barage and Sonawane, 2015). Moreover, in APP/PS1 transgenic mice, coexpression of APP and PS1 increased the level of Aβ_42_ but not that of Aβ_40_ (Jankowsky et al., 2004). In our study, we analyzed the levels of hippocampal and frontal Aβ_42_ reacted with monomeric, oligomeric and fibrillar forms of beta amyloid_1–42_ peptide. The Aβ_42_ can recognize humans and mice. We found that the Aβ_42_ level in the AD group was significantly higher than in the ADT group in the frontal and hippocampus (Figure 6). Aβ_42_ is highly neurotoxic and can cause prominent loss and neuronal damage (Perl, 2010). Significantly increased Aβ_42_ in the AD group caused the AD group to experience more severe neurological damage than the ADT group. This may be the reason for the significant differences in spatial learning and memory between the AD group and the ADT group. It also directly showed that AtDCS treatment significantly downregulated the level of Aβ_42_ in the brains of the early-stage APP/PS1 transgenic mice. This evidence can be used as direct support for the treatment of early-stage AD disorders with AtDCS.

In addition, the levels of Aβ_42_ differed across brain regions of APP/PS1 transgenic mice. Consistent with a previous study (Xiong et al., 2011), in the brain regions examined, the levels of Aβ_42_ were higher in the frontal cortex than in the hippocampus. The AD group had a higher level of Aβ_42_ in these two regions than did the ADT group, which may be the cause of the poor spatial learning and memory observed in the MWM in the AD group. Our results suggest that deposition of different Aβ_42_ levels in different brain regions may have different effects on the central nervous system and behavioral performance. Different levels of Aβ_42_ in the brain may be closely related to neural signal transmission. The hippocampus is an important part of the brain that is closely related to learning and memory. The trisynaptic loop, the hippocampus-prefrontal neural circuit, and other pathways are involved in the learning and memory processing of the hippocampus (Izaki et al., 2008; Brewer et al., 2013; Knierim, 2015). In our study, the AD group had higher Aβ_42_ levels than the ADT group in the DG and CA2-3 regions of the hippocampus (Figure 6B). The Aβ_42_ immunofluorescence results in the AD group showed that the number of Aβ_42_ deposits in the DG region was higher than that in the CA2-3 region (Figure 6C). The neurotoxicity of high levels of Aβ_42_ causes severe damage to the hippocampal DG and CA2-3. In the ADT group treated with AtDCS, a low level of Aβ_42_ was found scattered in the DG and CA2-3 regions (Figure 6C). The activity of DG in the hippocampus affects spatial memory (Brewer et al., 2013; Bui et al., 2018). The CA3 of the hippocampus is also important for spatial memory processes, especially the memory consolidation of spatial information (Florian and Roullet, 2004). As a result, severe spatial learning and memory disorders have occurred in AD mice. The mice in the ADT group exhibited a shorter escape latency and path length in the hidden platform experiments and more platform-region crossings and a longer time spent in the third quadrant in the probe test than the mice in the AD group (Figure 3). In addition, low levels of Aβ_42_ appeared in the CA1 region of the AD group (Figure 6Ck). These results potentially indicated that the spread of Aβ follows the pathway of neural signaling, thereby gradually invading the AD brain. We speculate that AtDCS may slow the neurotoxic invasion of Aβ_42_, thereby slowing the progression of AD.

Compared with the CTL group, the AD group and the ADT group had different expression levels of GFAP, indicating that the AD group and the ADT mice had different degrees of inflammatory abnormalities, consistent with our previous studies (Yu et al., 2015; Yang et al., 2019). Neuroinflammation is an early pathological manifestation in the AD brain and is a basic protective immune response in the central nervous system (Eikelenboom et al., 2010). The inflammatory response in AD is manifested by the activation of microglia and astrocytes. In recent years, astrocytes have been found to play an increasingly important role in AD. The early appearance of Aβ plaques in APP/PS1 transgenic mice at 3 months of age is accompanied by the appearance of GFAP (Zhu et al., 2017). Neurotoxic Aβ oligomers induce astrocyte activation (Nitta et al., 1997). Activated astrocytes are dysfunctional, undergo an inflammatory cascade, and abnormally release γ-aminobutyric acid (γ-GABA; Jo et al., 2014), affecting the glutamate cycle (Olabarria et al., 2011), which dysregulates Ca^2 +^ homeostasis and signaling and promotes Aβ deposition and synaptic plasticity damage in APP/PS1 mice in the early stage of AD progression (Gómez-Gonzalo et al., 2017). Therefore, the level of GFAP expression in the AD group was significantly higher than that in the CTL group (Figures 5C, 7B). In the ADT group treated with AtDCS, GFAP expression levels were decreased, and Aβ_42_ was surrounded by activated astrocytes (Figure 7C), which is consistent with previous studies (Itagaki et al., 1989; Yu et al., 2015; Zhu et al., 2017; Yang et al., 2019). However, AtDCS increases glial activation in the early stages and decreases with time (Rueger et al., 2012; Pikhovych et al., 2016). With high levels of activated glial cells, glial cells express renin (NEP), insulin degrading enzyme (IDE), endothelin converting enzyme (ECE), angiotensin converting enzyme (ACE), matrix metalloproteinase-9 and 2 (MMP-9, MMP-2) and other Aβ-degrading enzymes, and low density lipoprotein receptor related protein 1 (LRP1), scavenger receptor B1 (SCARB1) and RAGE transporters such as receptors for advanced glycation end products promote the degradation and clearance of Aβ, thereby reducing neurotoxicity and reducing the activation of glial cells and inflammation (Farris et al., 2003; Yan et al., 2006; Mulder et al., 2012; Li et al., 2014). In addition, AtDCS can continuously depolarize astrocyte transmembrane potentials and alter ion channel function to improve Ca^2+^ levels (Nitsche et al., 2003; Stagg et al., 2009) and regulate neurotransmitter (glutamate, γ-GABA) balance (Rueger et al., 2012; Ruohonen and Karhu, 2012), promoting normalization of glial function and reducing inflammation. Our detection of GFAP expression levels occurred at the end of the behavioral test, in which glial cells and reduced inflammation were detected. Overall, AtDCS ultimately downregulated the expression level of GFAP to improve inflammation in AD. GFAP expression is downregulated, reducing the overexpression of inflammatory factors, dysregulation of Ca^2+^ and abnormalities of glutamate and GABA, thereby reducing the number of Aβ deposits, inhibiting Aβ neurotoxicity. The may be one of the mechanisms of action of AtDCS in early-stage AD. We hypothesize that another pathway by which the mechanisms of action of AtDCS in early-stage AD is that AtDCS may directly affect production and degradation of Aβ, thereby attenuating neurotoxicity and reducing GFAP activation. APP is cleaved by β-secretase and γ-secretase, and then produce Aβ in amyloidogenic pathway. Another proteolytic processing pathway is non-amyloidogenic pathway in which APP is cleaved by α-secretase and γ-secretase (Haass et al., 2012; Barage and Sonawane, 2015). In the specimens of AD patients and the APP/PS1 double transgenic mouse model, the α-secretase of the non-amyloidogenic pathway of APP is decreased, and the β-secretase of the amyloidogenic pathway of APP is increased, and the Aβ-degrading enzyme is decreased, which could lead to an imbalance in the production and degradation of Aβ (Holsinger et al., 2002; Kummer et al., 2012; Sogorb-Esteve et al., 2018; Yang et al., 2018; Sun et al., 2019b). Our results showed that Aβ_42_ level in the ADT group was significantly lower than that in the AD group. Therefore, AtDCS may directly affect the level of α-secretase, β-secretase and Aβ-degrading enzyme, reduce the proteolytic processing pathway of APP in amyloidogenic pathway, increase the proteolytic processing pathway of APP in non-amyloidogenic pathway and degradation of Aβ, and thus reduce the level of Aβ. A decrease in Aβ reduces the neurotoxicity of Aβ, which in turn attenuates GFAP activation and the inflammatory cascade. A decrease in the neurotoxicity of Aβ reduces neuronal damage. NF200 immunohistochemistry revealed that the expression level of NF200 in the ADT group was significantly higher than that in the AD group, and neuronal damage in the AD group was severe. Nissl staining also showed that Nissl bodies of the ADT group were more neatly arranged and clearly visible than those of the AD group, indicating that the neuronal damage in the early-stage APP/PS1 transgenic mice was effectively improved by AtDCS treatment.

The safety of tDCS parameters is a factor that cannot be ignored. Currently, the safety of tDCS tends to be assessed by charge density (current intensity*duration/electrode size; Truong and Bikson, 2018). The charge density of tDCS was 128.571 kC/m^2^ (500 μA, 3.5 mm^2^, 15 min) and did not affect rat brain tissues or the cortex (Rueger et al., 2012). When tDCS was applied to the mouse cortex with a charge density of 99 kC/m^2^ (500 μA, 2.27 mm^2^, 15 min), the cortex of the mouse was intact. However, when the charge density was 198 kC/m^2^ (250 μA, 2.27 mm^2^, 15 min), the neuronal integrity of 50% of the mouse cortex was destroyed (Pikhovych et al., 2016). In humans, the charge intensity of tDCS does not exceed 1.44 kC/m^2^ (current intensity: 1–2 mA, electrode size: 25 cm^2^–35 cm^2^, duration: 10–30 min; Stagg and Nitsche, 2011; Berryhill and Martin, 2018; Truong and Bikson, 2018), which is far lower than the parameters applied to animals (Nitsche and Paulus, 2011). Animal and human tDCS parameters cannot be compared as a reference. In this study, we focused on the study of tDCS in learning and memory in AD model mice, which showed the same characteristics as those of tDCS in patients with AD. Therefore, tDCS can be considered safe as long as the charge density of tDCS is controlled within a reasonable range. The tDCS charge density used in this study was 85.987 kC/m^2^ (150 μA, 3.14 mm^2^, 30 min), which was below 99 kC/m^2^, and tDCS was considered safe for APP/PS1 mice.

The results of this study open up a new path for the early-stage treatment of AD. The course of AD is long, and AtDCS may be an effective intervention in the early stage of AD. AtDCS is a safe, economical, and well-tolerated treatment for the course of AD (Prehn and Fläel, 2015). Our research is still in its infancy, and further research is needed in the future. First, this study focused on the hippocampus. The entorhinal cortex is the first region of the brain affected in AD (Khan et al., 2014) and should be further explored. In addition, there is a close relationship between the prefrontal cortex and the hippocampus (Izaki et al., 2008) that requires further exploration. Second, there is now a new 5xFAD AD mouse model that better reflects the course of AD than the APP/PS1 mouse model, allowing for a deeper study of AD pathology. Third, in-depth studies on enzymes associated with Aβ generation or degradation, calcium signals and neurotransmitters, such as glutamate and γ-GABA, can performed through experimental methods such as *in vivo* and *in vitro* optogenetic and patch clamp techniques. In addition, the longitudinal effects and the mechanisms of action of AtDCS in APP/PS1 transgenic mice is also worth exploring.

## Conclusion

The 6-month-old APP/PS1 transgenic mouse model showed significant spatial learning and memory impairment, but there was no significant recognition memory impairment. Early intervention with AtDCS improved spatial learning and memory in the APP/PS1 transgenic mice at 6 months of age, reduced Aβ_42_ burden, and protected neurons. AtDCS could improve AD-related symptoms by activating many glial cells to promote the degradation and clearance of Aβ or directly affecting production and degradation of Aβ to reduce glial activation. Our results suggest that AtDCS is an effective means of early intervention in the early stage of AD.

## Data Availability Statement

The datasets generated for this study are available on request to the corresponding author.

## Ethics Statement

The animal study was reviewed and approved by the Laboratory Animal Welfare and Ethics Committee of the Army Medical University.

## Author Contributions

YL, WY, HW and XT conceived and designed the research and interpreted the results of the experiments. YL, NL, XY and CW performed the experiments. YL, BZ, WH and XW participated in data analyses and arranged the figures. YL and HW drafted the manuscript. All authors edited and revised the manuscript, approved the final manuscript and agreed to be accountable for all aspects of the work.

## Conflict of Interest

The authors declare that the research was conducted in the absence of any commercial or financial relationships that could be construed as a potential conflict of interest.
